# AK050834 Deficiency Prevents Vascular Aging via Opposite Regulation of Two Senescence‐Sensitive Genes ATF3 and p21

**DOI:** 10.1111/acel.70670

**Published:** 2026-08-18

**Authors:** Haoran Wu, Ping Yang, Yang Liu, Qiuting Li, Xinglin Wang, Linyi Tang, Liyun Li, Ting Huang, Xin Zhang, Chunxiang Zhang

**Affiliations:** ^1^ Department of Cardiology, Key Laboratory of Medical Electrophysiology, Ministry of Education, Nucleic Acid Medicine of Luzhou Key Laboratory, Institute of Cardiovascular Research, Basic Medicine Research Innovation Center for Cardiometabolic Diseases, Ministry of Education The Affiliated Hospital, Southwest Medical University Luzhou Sichuan China; ^2^ Department of Rehabilitation Medicine The Affiliated Hospital, Southwest Medical University Luzhou China; ^3^ School of Basic Medical Sciences Southwest Medical University Luzhou China

**Keywords:** AK050834, lncRNA, senescence, smooth muscle cells, vascular aging

## Abstract

Vascular aging, not only a victim of vascular cell senescence but also a driving hub of systemic aging, is a complex process in which both anti‐aging and pro‐aging proteins are involved. Thus, the ideal anti‐aging medication should have multiple protein targets; in this case, an lncRNA might be a good choice. AK050834 is a conserved lncRNA, but its roles in vascular biology and vascular aging are currently unknown. Here, we identified that AK050834 was highly expressed in vascular walls and in vascular smooth muscle cells (VSMCs). Notably, AK050834 expression was remarkably increased in aged vessels. By using gain‐of‐function, loss‐of‐function, and gene knockout approaches, we identified that AK050834 is a strong regulator of VSMC senescence and vascular aging. In terms of the mechanisms, we found that AK050834 suppressed the expression of the anti‐aging protein ATF3 via direct interaction with its promoter, which was further confirmed by a rescue experiment. In addition, AK050834 increased the expression of the pro‐aging protein p21 by stabilizing its mRNA. Finally, we found that AK050834 deficiency could effectively prevent vascular aging in mice in vivo. The interesting dual and opposite effects of AK050834 on the anti‐aging protein ATF3 and the pro‐aging protein p21 suggest that AK050834 might be a novel diagnostic marker as well as an ideal therapeutic and preventive target for vascular aging and many related diseases.

## Graphical abstract

Schematic diagram illustrating the molecular mechanism by which lncRNA AK050834 drives vascular smooth muscle cell (VSMC) senescence. Extracellular stimuli, including D‐galactose and natural aging, induce the upregulation of AK050834. AK050834 exerts dual regulatory functions: (1) In the nucleus, it directly binds to the ATF3 promoter region to suppress its transcriptional activity; (2) In the cytoplasm, it interacts with p21 mRNA to prevent its degradation, thereby promoting the high translation and expression of p21. The consequent downregulation of ATF3 and upregulation of p21 collectively activate a downstream pathogenic driver, ultimately resulting in VSMC senescence, as characterized by elevated SA‐β‐gal activity, DNA damage (γ‐H2AX), and reactive oxygen species (ROS) accumulation. Importantly, exogenous ATF3 supplementation functions as a rescue feedback loop to block this AK050834‐ATF3‐p21 signaling axis and effectively prevent the senescent phenotype.

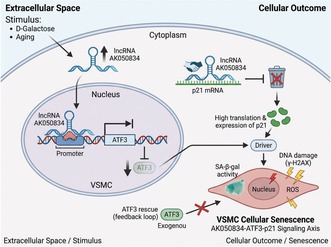

## 1 Introduction

Vascular aging, characterized by functional deterioration and pathological structural remodeling, is not only a consequence of vascular cell senescence but also a “driving hub” of systemic aging of the whole body, as described by the famous medical saying, “A man is as old as his arteries” (Kleefeldt et al. 2019). As populations across the globe are experiencing an accelerated process of aging, there has been a marked increase in the prevalence of vascular aging‐related diseases, including hypertension, atherosclerosis, and heart failure (Luo et al. 2021). These conditions have become significant causes of mortality and disability among the elderly (Lacolley et al. 2025). Therefore, research on anti‐vascular aging is extremely important and urgent.

It is well known that aging is a complex process in which multiple proteins are involved, as demonstrated by many proteomics studies. Among them, some proteins are pro‐aging proteins, such as p53 and p21, whereas others are anti‐aging proteins, such as activator transcription factor 3 (ATF3) (Basilicata et al. 2025; Picos et al. 2025; Nie et al. 2025). Thus, targeting a single age‐related protein for anti‐aging might be less effective and may not be reasonable. In contrast, ideal anti‐aging medications should target multiple key age‐related proteins. In this context, non‐coding RNAs might be good candidates because they can modulate the expression of multiple proteins at different levels (He et al. 2025). The acknowledgment of non‐coding RNAs through the awarding of the 2024 Nobel Prize in Physiology or Medicine, coupled with the clinical application of RNA‐targeting therapeutics—exemplified by inclisiran, a biannual siRNA treatment for hypercholesterolemia—highlights their therapeutic significance. However, all currently identified aging‐related non‐coding RNAs have been found to regulate either pro‐aging protein(s) or anti‐aging protein(s) (Turko et al. 2025).

AK050834 was originally identified as one of the most abundant long non‐coding RNAs (lncRNAs) in human monocytes and exhibits high evolutionary conservation between humans and mice (Mirsafian et al. 2016). However, its biological function in the cardiovascular system remains largely unexplored. In the present study, we discovered that AK050834 is highly expressed in vascular walls and is predominantly enriched in vascular smooth muscle cells (VSMCs). Mechanistically, we revealed for the first time that AK050834 exerts a dual pro‐senescence role: it directly suppresses the transcription of the anti‐aging transcription factor ATF3 by binding to its promoter, while simultaneously stabilizing the pro‐aging factor p21 mRNA through its 3′ untranslated region (3′UTR). Collectively, these findings establish AK050834 as a critical driver of vascular aging, suggesting that it may represent a promising intervention target for age‐related vascular diseases.

## 2 Materials and Methods

### 2.1 Animals and Anesthetics and Analgesia Statement

All animal procedures were approved by the Institutional Animal Care and Use Committee (IACUC) of Southwest Medical University (Approval No.: 20241028‐002) and were performed in accordance with the Guide for the Care and Use of Laboratory Animals (National Institutes of Health, USA). Male C57BL/6J mice were used in this study. For the naturally aged mouse model, 18‐month‐old mice were designated as the aged group, whereas 8‐week‐old mice served as the young controls. For the D‐galactose (D‐gal)‐induced aging model, mice were treated with D‐gal at a dose of 500 mg/kg/day for 8 weeks via intraperitoneal injection. All mice were anesthetized using isoflurane (inspired concentration of 1.5%–3%) and subsequently euthanized by increasing the isoflurane concentration to 5%, after which aortas were harvested.

### 2.2 Generation of lncRNA AK050834 Knockout Mice

LncRNA AK050834 knockout (AK‐KO) mice were generated on a C57BL/6J background using the CRISPR/Cas9 system. Single‐guide RNAs (sgRNAs) targeting exon regions of the lncRNA AK050834 gene were designed and co‐injected with Cas9 mRNA into fertilized mouse eggs. Founder mice (F0) were genotyped by PCR amplification of the target locus followed by Sanger sequencing. Heterozygous (AK050834^+^/^−^) offspring from founder crosses were interbred to generate homozygous AK‐KO (AK050834^−^/^−^) mice. Wild‐type (WT) littermates were used as controls. Mice were housed under specific pathogen‐free (SPF) conditions with a 12‐h light/dark cycle and free access to food and water.

### 2.3 Cell Culture and Treatments

The human aortic vascular smooth muscle cells (HA‐VSMCs) and HEK‐293 T cells used in this study were obtained from the American Type Culture Collection (ATCC). HA‐VSMCs were cultured in Dulbecco's Modified Eagle Medium (DMEM), while HEK‐293 T cells were cultured in RPMI‐1640 medium. Both media were supplemented with 10% fetal bovine serum (FBS) and 1% penicillin–streptomycin. Cells were maintained in a humidified incubator at 37°C with 5% CO_2_, and the medium was refreshed every 2 to 3 days. For senescence induction, cells were treated with D‐galactose (D‐gal; Solarbio, Catalog No.: D8310) at a final concentration of 40 g/L for 24 h. For overexpression or knockdown experiments, cells were transfected with an AK050834‐expressing vector (constructed in pCS2+) or specific siRNA (designed and synthesized by Shanghai Genepharma) using Lipofectamine 3000 (Invitrogen) according to the manufacturer's protocol. ATF3 overexpression was achieved by transfection with a human ATF3 expression plasmid (MiaoLingBio, Plasmid #P85304).

### 2.4 RNA Extraction and RT‐qPCR

Total RNA was extracted from cells or homogenized mouse aortic tissues using TRIzol reagent (Vazyme, R401‐01). cDNA was synthesized from 1 μg of total RNA using a reverse transcription kit (Vazyme, R433‐01). Quantitative PCR (qPCR) was performed using SYBR Green Master Mix (Vazyme, Q712‐02) on a QuantStudio 5 Real‐Time PCR System (Applied Biosystems). The reaction conditions were: 95°C for 5 min, followed by 40 cycles of 95°C for 15 s and 60°C for 30 s. Gene expression levels were calculated using the 2^−ΔΔ^Ct method and normalized to *ACTB*.

### 2.5 Protein Extraction and Western Blotting

Cells or tissues were lysed in RIPA buffer (Solarbio, R0010) containing protease and phosphatase inhibitors. Protein concentrations were determined using a BCA assay kit (Thermo Fisher Scientific, A55861). Equal amounts of protein (20–30 μg per lane) were separated by 10% or 12.5% SDS‐PAGE and transferred to PVDF membranes (Millipore). After blocking with 5% non‐fat milk, membranes were incubated overnight at 4°C with primary antibodies against ATF3 (1:1000, Abcam, ab207434), P53 (1:1000, Cell Signaling Technology, 2524), P21 (1:500, Abcam, ab109520), P16 (1:2000, Abcam, ab211542), γ‐H2AX (1:1000, Cell Signaling Technology, 9718), or β‐ACTIN (1:20000, Proteintech, Cat NO. 66009‐1‐lg). Membranes were then incubated with HRP‐conjugated secondary antibodies (1:5000, Jackson ImmunoResearch) and visualized using an ECL detection system (Bio‐Rad).

### 2.6 Senescence‐Associated β‐Galactosidase (SA‐β‐Gal) Staining

SA‐β‐gal staining was performed using a senescence detection kit (Beyotime, C0602) according to the manufacturer's instructions. For cells, after treatment, they were fixed and incubated with X‐Gal staining solution at 37°C overnight (without CO_2_). For frozen tissue sections, sections were fixed and stained similarly. Senescent cells (blue staining) were observed under an optical microscope and quantified from five random fields per sample.

### 2.7 Histological Analysis and Quantification

Aortic tissues were fixed in 4% paraformaldehyde, embedded in paraffin, and sectioned at 4–5 μm thickness. The sections were then subjected to Hematoxylin and Eosin (H&E), Masson's trichrome, and Eosin staining according to the manufacturer's protocols. Images were captured using a light microscope (Olympus, Japan). For quantitative analysis, ImageJ software (National Institutes of Health, USA) was employed. Inflammatory cell infiltration was evaluated in H&E‐stained sections by randomly selecting at least 5 high‐power fields per section. The total number of infiltrated inflammatory cells was manually counted using the “Cell Counter” plugin in ImageJ and normalized to the tissue area. Collagen volume fraction was quantified in Masson's trichrome‐stained sections by using the “Color Threshold” function to distinguish the blue‐stained collagen fibers from the red‐stained cytoplasm and blood cells. The collagen area was calculated as the percentage of the total tissue area. Eosinophil percentage was determined in Eosin‐stained sections by randomly selecting 3–5 fields. Cells with characteristic bright pink cytoplasmic granules were identified as eosinophils. The percentage was calculated as the number of eosinophils divided by the total number of nucleated cells.

### 2.8 Immunofluorescence and ROS Staining

For immunofluorescence, cells or frozen tissue sections were fixed, permeabilized, and blocked with 5% BSA. Samples were incubated with primary antibody against γ‐H2AX (1:500, Cell Signaling Technology, 9718) overnight at 4°C, followed by incubation with Alexa Fluor 488‐conjugated secondary antibody (1:200, Invitrogen). Nuclei were counterstained with DAPI (Beyotime, P0131). For ROS detection, cells or tissue sections were incubated with 10 μM DCFH‐DA (Beyotime, S0033S) at 37°C for 30 min. Fluorescence images were captured using a confocal microscope (Zeiss LSM 880), and fluorescence intensity was quantified using ImageJ software.

### 2.9 Cell Functional Assays

#### 2.9.1 CCK‐8 Assay

Cell viability was assessed using Cell Counting Kit‐8 (CCK‐8; Beyotime, C0039). Cells were seeded in 96‐well plates (1 × 10^4^ cells/well), and 10 μL of CCK‐8 solution was added to each well. After incubation for 2 h, absorbance at 450 nm was measured using a microplate reader.

#### 2.9.2 EdU Assay

Cell proliferation was evaluated using the Click‐iT EdU Imaging Kit (Vazyme, A411‐01). Cells were incubated with 10 μM EdU for 2 h, fixed, and stained according to the kit protocol. EdU‐positive cells were counted under a fluorescence microscope.

#### 2.9.3 Transwell Migration Assay

Cells (5 × 10^4^) in serum‐free medium were plated into the upper chamber of a Transwell insert (8‐μm pore; Corning). The lower chamber contained medium with 10% FBS. After 24 h, migrated cells on the lower membrane surface were fixed, stained with crystal violet, and counted.

#### 2.9.4 Cell Migration Assay via Scratch

Cells were grown to 100% confluence in 6‐well plates. A scratch was made using a sterile 200 μL pipette tip. Images were taken at 12 h and 24 h, and the migration distance was analyzed using ImageJ.

#### 2.9.5 Cell Cycle Analysis

Cells were fixed in 70% ethanol, treated with RNase A, and stained with propidium iodide (PI; 50 μg/mL). DNA content was analyzed by flow cytometry (BD FACS Verse), and cell cycle distribution was determined using ModFit LT software.

### 2.10 RNA Sequencing and Bioinformatic Analysis

Total RNA was extracted from aortic tissues of WT and AK‐KO mice. mRNA sequencing service was provided by CloudSeq Inc. (Shanghai, China). Briefly, total RNA was extracted with Trizol (Invitrogen, Carlsbad, CA, USA) and subjected to mRNA isolation using GenSeq mRNA Purification Kit (GenSeq Inc., Shanghai, China). Then, the purified mRNA was used for library construction with GenSeq Directional RNA Library Prep Kit (GenSeq Inc., Shanghai, China) by following manufacturer's recommendations. The mRNA was fragmented into ~300 nt in length. The first‐strand cDNA was synthesized from the RNA fragments by reverse transcriptase and random hexamer primers, and the second‐strand cDNA was synthesized in 2nd Strand Synthesis Buffer with dUTP Mix. Then, the double‐stranded cDNA fragments were subjected to end‐repair and dA‐tailing, followed by adapter ligation. The adapter‐ligated DNA samples were PCR amplified and purified to obtain sequencing libraries. Finally, the libraries were sequenced with sequencer on the paired‐end 150 bp mode.

Paired‐end reads were obtained from sequencing. fastp software (v0.23.4) was used for data preprocessing, including quality control (Q30) and adaptor‐trimming processing, from which high‐ quality reads were obtained. High‐quality reads were aligned to the reference genome using HISAT2 software (v2.2.1). Gene level raw counts were then obtained as mRNA expression profiles using feature Count software (v2.0.6). Fold change (FC) and *p*‐value between the two sets of samples were normalized and calculated using edge R software (v4.0.16). After getting differentially expressed (DE) mRNAs by filtering FC and *p*‐value, the DE mRNAs were used for GO functional analysis, KEGG and GSEA pathway analysis.

### 2.11 Fluorescence in Situ Hybridization (FISH)

The subcellular localization of AK050834 was determined using a FISH kit (RiboBio) according to the manufacturer's protocol. Briefly, cells grown on coverslips or frozen tissue sections were fixed with 4% paraformaldehyde, permeabilized with 0.5% Triton X‐100, and pre‐hybridized. Then, a specific fluorescently labeled probe targeting AK050834 was hybridized overnight at 37°C in a dark humidified chamber. After washing, the nuclei were counterstained with DAPI. Images were captured using a confocal laser scanning microscope.

### 2.12 AK050834 Target Prediction

The potential for AK050834 to form DNA–RNA triplex structures with the ATF3 promoter region was predicted using the online tool LongTarget (http://www.gaemons.net/LongTarget). The analysis was based on the sequence complementarity and energy stability rules for triplex formation.

### 2.13 Luciferase Reporter Assay

A fragment of the ATF3 promoter was cloned into the pGL3‐Basic luciferase reporter vector. HEK‐293 T cells were co‐transfected with the reporter construct, a *Renilla* luciferase control vector (pRL‐TK) or an AK050834 overexpressed plasmid. After 48 h, firefly and *Renilla* luciferase activities were measured using a Dual‐Luciferase Reporter Assay System. Firefly luciferase activity was normalized to *Renilla* activity.

### 2.14 Actinomycin D Chase Assay

HEK‐293 T were treated with Actinomycin D (5 μg/mL) to inhibit transcription. Cells were harvested at 0, 2, 4, 6, and 8 h post‐treatment. The mRNA levels of p21 and a stable internal control (ACTB) were measured by RT‐qPCR. The half‐life (t_₁/2_) of p21 mRNA was calculated based on the decay curve.

### 2.15 Chromatin Isolation by RNA Purification (ChIRP) Assay

Formaldehyde‐crosslinked cells were lysed and sonicated to shear chromatin into 100–500 bp fragments. The cleared lysate was divided into two aliquots and incubated overnight at 37°C with biotinylated antisense oligonucleotide pools (Odd and Even) in hybridization buffer. Probe–chromatin complexes were captured using streptavidin magnetic beads, washed thoroughly with wash buffer at 37°C, and eluted. Bead‐bound DNA was digested with RNase A, treated with Proteinase K, and purified by phenol–chloroform extraction and ethanol precipitation. An aliquot of sonicated chromatin was reserved as Input control and processed identically. Enrichment was calculated as % Input = 2^(Ct_Input − Ct_IP) × (1/17) × 100%, where 1/17 corrects for the Input dilution factor. Three technical replicates were analyzed per sample; data are shown as mean ± SD. Significance between loci was assessed by unpaired two‐tailed Student's *t*‐test (*p* < 0.05 considered significant). The primers used for detecting the candidate binding sites were as follows:

- Site 1 (chr1:212563902–212,563,950): Forward 5′‐CACCTGCCAACACGCCAA‐3′, Reverse 5′‐GGCGACTGTAACACAATGGT‐3′ (amplicon size: 223 bp);
- Site 2 (chr1:212564461–212,564,512): Forward 5′‐TCTTGCTGTTAAGTGACGAATG‐3′, Reverse 5′‐ACTGGCACGTTTGTTACAATCA‐3′ (amplicon size: 301 bp).

### 2.16 Statistical Analysis

All data are presented as mean ± standard deviation (SD). Statistical analyzes were performed using GraphPad Prism 9.0. Comparisons between two groups were analyzed by Student's *t*‐test. Comparisons among multiple groups were analyzed by one‐way analysis of variance (ANOVA) followed by Tukey's post hoc test. A *p*‐value < 0.05 was considered statistically significant.

## 3 Results

**1. The Long non‐coding RNA AK050834 is highly expressed in vascular walls and in VSMCs**.

To elucidate the expression pattern of AK050834 (hereafter termed AK), we employed real‐time quantitative reverse transcription polymerase chain reaction (RT‐qPCR) to analyze its expression levels in organs from 8‐week‐old C57 mice. The results indicated high AK expression in the heart, lung, and aorta (Figure 1A). Subsequent analysis of the expression levels of this lncRNA in various vascular cell types revealed its high expression in monocyte‐derived macrophages (THP‐1), aortic smooth muscle cells (HA‐VSMC), and aortic endothelial cells (HAEC), but low expression in fibroblasts (HAF) and lymphatic endothelial cells (HLEC) (Figure 1B). Given that VSMCs exhibited the highest expression among the examined vascular cells, the present study focused on VSMCs to investigate the potential roles of AK in vascular senescence. Since the biological function of lncRNAs is contingent upon their cellular localization (Yang et al. 2018), we employed a FISH experiment to investigate AK's localization within aortic VSMCs. The results demonstrated that the highest levels of AK were located within the nuclei, with low levels in the cytoplasm (Figure 1C). Additionally, homology analysis revealed a sequence identity of 91.4% between human AK and mouse AK (Figure S1), indicating that AK is a conserved lncRNA between humans and mice.

**FIGURE 1:**
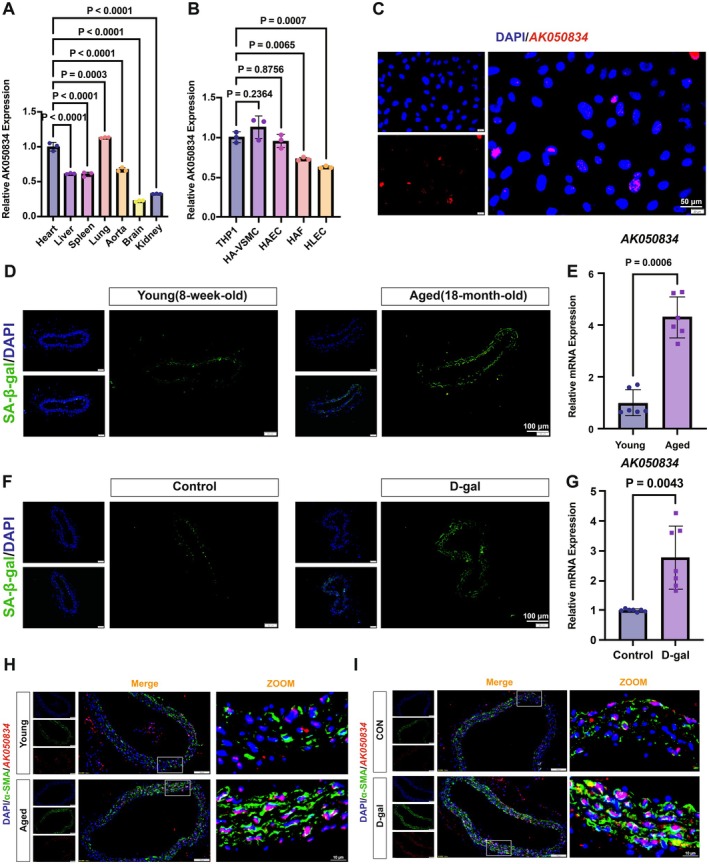
The expression of AK050834 and its changes in aged vessels. (A) The relative mRNA expression levels of AK050834 across major organs (heart, liver, spleen, lung, aorta, brain, and kidney) from 8‐week‐old C57BL/6J mice. (B) Relative mRNA expression levels of AK050834 in different vascular cell types, including monocyte‐derived macrophages (THP‐1), human aortic vascular smooth muscle cells (HA‐VSMCs), human aortic endothelial cells (HAECs), human aortic fibroblasts (HAFs), and human lymphatic endothelial cells (HLECs). (C) Subcellular localization of AK050834 in HA‐VSMCs detected by RNA fluorescence in situ hybridization (FISH), with nuclei counterstained with DAPI (blue). Scale bar, 50 μm. (D) Representative immunofluorescence images of SA‐β‐gal (green) and DAPI (blue) staining in aortas from naturally aged mice and their young controls. Scale bar, 100 μm. (E) Relative mRNA expression levels of AK050834 in aortas from naturally aged mice (18‐month‐old) and their young controls (8‐week‐old). *n* = 6. (F) Representative immunofluorescence images of SA‐β‐gal (green) and DAPI (blue) staining in aortas from D‐galactose (D‐gal)‐induced aging mice and their controls. Scale bar, 100 μm. (G) Relative mRNA expression levels of AK050834 in aortas from D‐galactose (D‐gal)‐induced aging mice and their controls. *n* = 7. (H) Representative co‐immunofluorescence images showing the localization of AK050834 (red) and α‐SMA (green) in aortas from young (8‐week‐old) and naturally aged mice (18‐month‐old), with nuclei stained with DAPI (blue). The right panels show the zoomed‐in views of the indicated areas. Scale bar, 10 μm (I) Representative co‐immunofluorescence images showing the localization of AK050834 (red) and α‐SMA (green) in aortas from control and D‐gal‐induced aging mice, with nuclei stained with DAPI (blue). The right panels show the zoomed‐in views of the indicated areas. Scale bar, 10 μm Data represent mean ± SEM. Comparisons between two groups were analyzed by Student's *t*‐test. Comparisons among multiple groups were analyzed by one‐way analysis of variance (ANOVA) followed by Tukey's post hoc test. Exact *p*‐values are indicated in the respective panels. Scale bars are indicated in the respective images.

To test the potential involvement of AK in vascular aging, we applied two in vivo models: aorta from naturally aged mice and aorta from D‐galactose (D‐gal)‐induced aging mice. Senescence‐associated β‐galactosidase (SA‐β‐gal) staining revealed overt vascular senescence in the aortic walls of both naturally aged mice and D‐gal‐induced mice when compared with their respective young control mice (Figure 1D,F). Notably, RT‐qPCR analysis demonstrated that the expression levels of AK in aortas from both aging models were remarkably increased compared with those in the control groups (Figure 1E,G). To further precisely pinpoint the cellular source of this elevated AK within the vascular wall, we performed co‐immunofluorescence staining for AK (red) and α‐smooth muscle actin (α‐SMA, green), a specific marker for VSMCs. The results revealed that AK signals predominantly co‐localized with α‐SMA‐positive cells in the tunica media of the aortic wall (Figure 1H). Importantly, this co‐localized AK signal in VSMCs was markedly intensified in the aortas from both naturally aged and D‐gal‐induced aging mice (Figure 1I). Collectively, these findings demonstrate that AK is a conserved, nuclear‐enriched lncRNA highly expressed in VSMCs, and its expression is significantly induced in the vascular wall during VSMC senescence.

**2. AK050834 overexpression exacerbates D‐galactose‐induced VSMC senescence**.

To test the potential role of AK in VSMC senescence, we first overexpressed it in HA‐VSMCs via an AK‐expressing plasmid (OE‐AK050834) and determined the effect of AK overexpression on D‐gal‐induced VSMC senescence. The results showed that AK overexpression increased the proportion of SA‐β‐gal‐positive cells in the D‐gal‐treated group (Figure 2A,B). Compared with the control group, cells overexpressing AK exhibited more pronounced senescent morphological features under D‐gal induction, including cell flattening and cytoplasmic enlargement. Immunofluorescence staining for the DNA damage marker γ‐H2AX revealed increased γ‐H2AX expression in AK‐overexpressing cells following D‐gal treatment (Figure 2C,D), suggesting heightened DNA damage. Concurrently, ROS fluorescence staining demonstrated that the overexpression of AK led to increased ROS production (Figure 2E,F). Subsequently, we conducted a more in‐depth examination of cellular senescence marker proteins at the molecular level. At the protein level, Western blot analysis revealed that overexpression of AK resulted in a significant increase in the expression of p53, p21, p16, and γ‐H2AX (Figure 2G,H). At the messenger RNA (mRNA) level, quantitative polymerase chain reaction (qPCR) validation confirmed the corresponding upregulation of these genes at the mRNA level (Figure S2). qPCR analysis of the senescence‐associated secretory phenotype (SASP) revealed that the expression levels of pro‐inflammatory factors, including *TNF‐α, IL‐1β, MCP‐1, and CXCL‐10*, in D‐galactose‐induced HA‐VSMCs were further elevated by AK overexpression (Figure 2I). These results suggest that AK is a strong pro‐senescence lncRNA in vascular cells.

**FIGURE 2:**
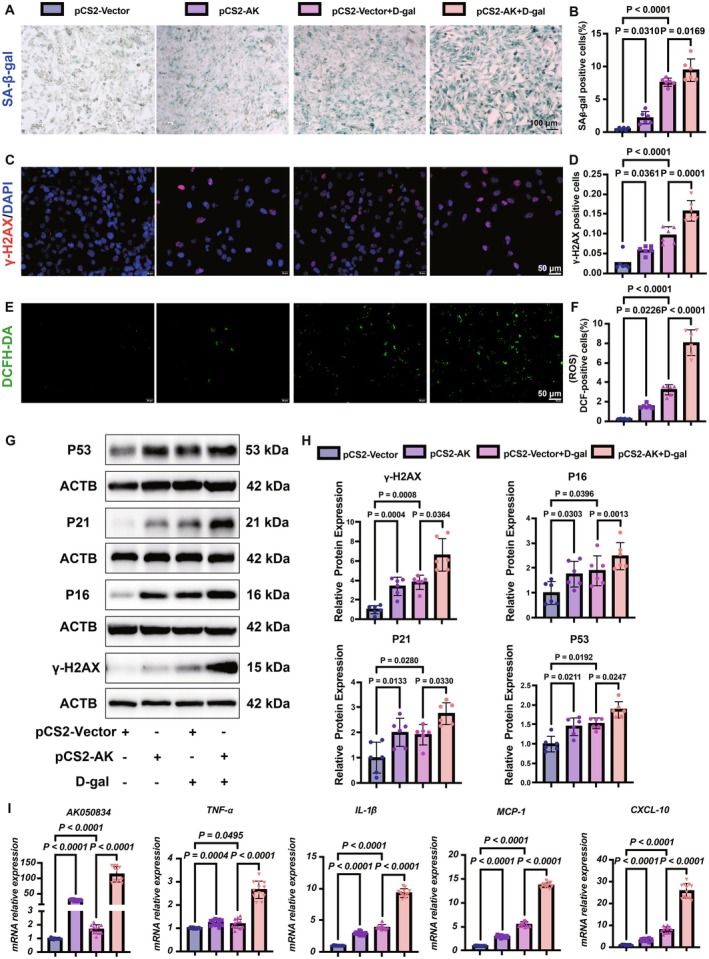
AK050834 overexpression exacerbates D‐galactose‐induced VSMC senescence. (A, B) Representative images (A) and quantitative analysis (B) of senescence‐associated β‐galactosidase (SA‐β‐gal) staining in HA‐VSMCs transfected with empty vector (pCS2‐Vector) or AK050834 overexpression plasmid (pCS2‐AK), followed by treatment with or without D‐galactose (D‐gal). *n* = 6. Scale bar, 100 μm. (C, D) Representative immunofluorescence images of γ‐H2AX (red) with DAPI (blue) staining (C) and quantification of γ‐H2AX‐positive nuclei (D) in the indicated groups. *n* = 6. Scale bar, 50 μm. (E, F) Representative images of DCFH‐DA fluorescence (green) for detecting reactive oxygen species (ROS) levels (E) and the percentage of ROS‐positive cells (F) in the indicated groups. *n* = 6. Scale bar, 50 μm. (G) Western blot analysis of the expression levels of senescence‐associated proteins (P53, P21, P16, and γ‐H2AX) and ACTB (internal control) in the indicated groups, with molecular weights indicated. (H) Quantification of relative protein expression of P53, P21, P16, and γ‐H2AX based on the Western blot results from panel G. *n* = 6. (I) Relative mRNA expression levels of AK050834 and senescence‐associated secretory phenotype (SASP) factors (TNF‐α, IL‐1β, MCP‐1, and CXCL‐10) determined by RT‐qPCR in the indicated groups. *n* = 12. Data are presented as mean ± SEM. Comparisons among multiple groups were analyzed by one‐way analysis of variance (ANOVA) followed by Tukey's post hoc test. Exact *p*‐values are indicated in the respective panels. Scale bars are indicated in the respective images.

**3. AK050834 knockdown alleviates D‐galactose‐induced VSMC senescence**.

SA‐β‐glucosidase staining was employed to confirm the effect of AK knockdown on D‐gal‐induced cellular senescence in HA‐VSMCs. The results showed that D‐gal‐induced senescence was inhibited by AK knockdown (Figure 3A,B). γ‐H2AX is a well‐known key molecule involved in mediating DNA damage and increasing oxidative stress during senescent processes. We found that AK knockdown could effectively block the increase of γ‐H2AX and ROS in D‐gal‐treated HA‐VSMCs (Figure 3C–F). Downregulation of AK led to a decrease in the expression of p53, p21, p16, and γ‐H2AX proteins in D‐gal‐induced VSMCs (Figure 3G,H), with comparable results at the mRNA level (Figure S3). qPCR analysis demonstrated that AK knockdown attenuated the expression of *TNF‐α, IL‐1β, MCP‐1, and CXCL‐10* (Figure 3I). These findings suggest that downregulation of AK could effectively prevent and treat VSMC senescence.

**FIGURE 3:**
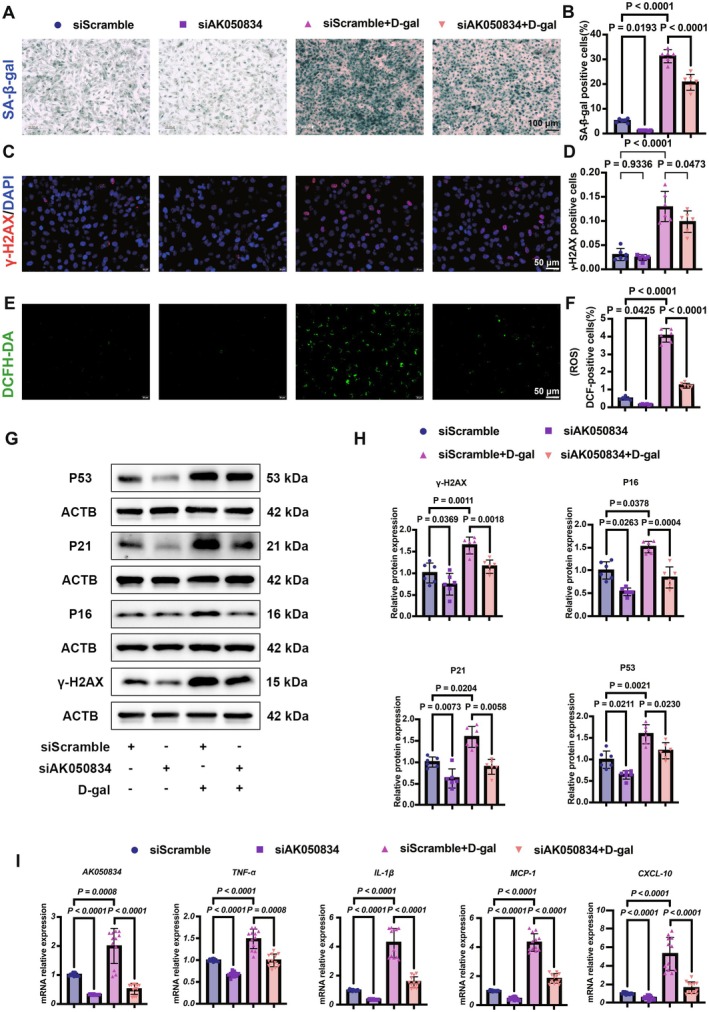
AK050834 knockdown alleviates D‐galactose‐induced VSMC senescence. (A, B) Representative images (A) and quantitative analysis (B) of senescence‐associated β‐galactosidase (SA‐β‐gal) staining in HA‐VSMCs transfected with scrambled siRNA (siScramble) or AK050834‐specific siRNA (siAK050834), followed by treatment with or without D‐galactose (D‐gal). Scale bar, 100 μm. *n* = 6 per group. (C, D) Representative immunofluorescence images of γ‐H2AX (red) with DAPI (blue) staining (C) and quantification of γ‐H2AX‐positive nuclei (D) in the indicated groups. Scale bar, 50 μm. *n* = 6 per group. (E, F) Representative images of DCFH‐DA fluorescence (green) for detecting reactive oxygen species (ROS) levels (E) and the percentage of ROS‐positive cells (F) in the indicated groups. Scale bar, 50 μm. *n* = 6 per group. (G) Western blot analysis of the expression levels of senescence‐associated proteins (P53, P21, P16, and γ‐H2AX) and ACTB (internal control) in HA‐VSMCs from the indicated groups, with molecular weights indicated. (H) Quantitative analysis of relative protein expression levels of γ‐H2AX, P16, P21, and P53 based on the Western blot results from panel G. *n* = 6 per group. (I) Relative mRNA expression levels of AK050834 and senescence‐associated secretory phenotype (SASP) factors (TNF‐α, IL‐1β, MCP‐1, and CXCL‐10) determined by RT‐qPCR in the indicated groups. *n* = 12 per group. Data are presented as mean ± SEM. Comparisons among multiple groups were analyzed by one‐way analysis of variance (ANOVA) followed by Tukey's post hoc test. Exact *p*‐values are indicated in the respective panels. Scale bars are indicated in the respective images.

**4. Knockout of AK050834 prevents vascular aging in mice in vivo**.

We demonstrated that AK was increased in aged aortas from mice and that AK was a critical regulator of vascular cell senescence in cultured VSMCs. We thus hypothesized that AK might play a key role in vascular aging in mice in vivo. To test this, we generated the AK050834 knockout mouse by employing CRISPR‐Cas9 technology. The relevant construction details and sgRNA target information are presented in Figure S4.

The initial approach was to utilize β‐galactosidase staining for the purpose of comparing vascular aging in the aortas of wild‐type naturally aged mice and AK‐KO naturally aged mice. The results demonstrated that AK‐KO markedly reduced the expression of β‐galactosidase within the vasculature (Figure 4A,B). p53 is a well‐established biomarker of aging. In this study, immunofluorescence assays were employed, using α‐SMA as an aortic smooth muscle marker, to detect p53 protein expression in aortic smooth muscle cells within the vascular walls. We found that AK‐KO attenuated p53 expression in aortic smooth muscle cells, confirming the capacity of AK‐KO to delay age‐related vascular senescence (Figure 4C,D). We then employed hematoxylin and eosin (H&E), Masson's trichrome, and Eosin staining to evaluate alterations in naturally aged vessels from wild‐type and AK‐KO mice. The results demonstrated that AK‐KO effectively mitigated age‐related inflammatory cell infiltration and collagen deposition within aged vessels (Figure 4E–H). Subsequently, we determined the molecular effects of AK‐KO on aging‐associated protein expression. The results showed that AK‐KO led to a reduction in the expression of p53, p16, p21, and γ‐H2AX, with a notable decrease in p21 expression (Figure 4I,J). The collective results demonstrated that AK deficiency could effectively inhibit vascular aging in mice in vivo.

**FIGURE 4:**
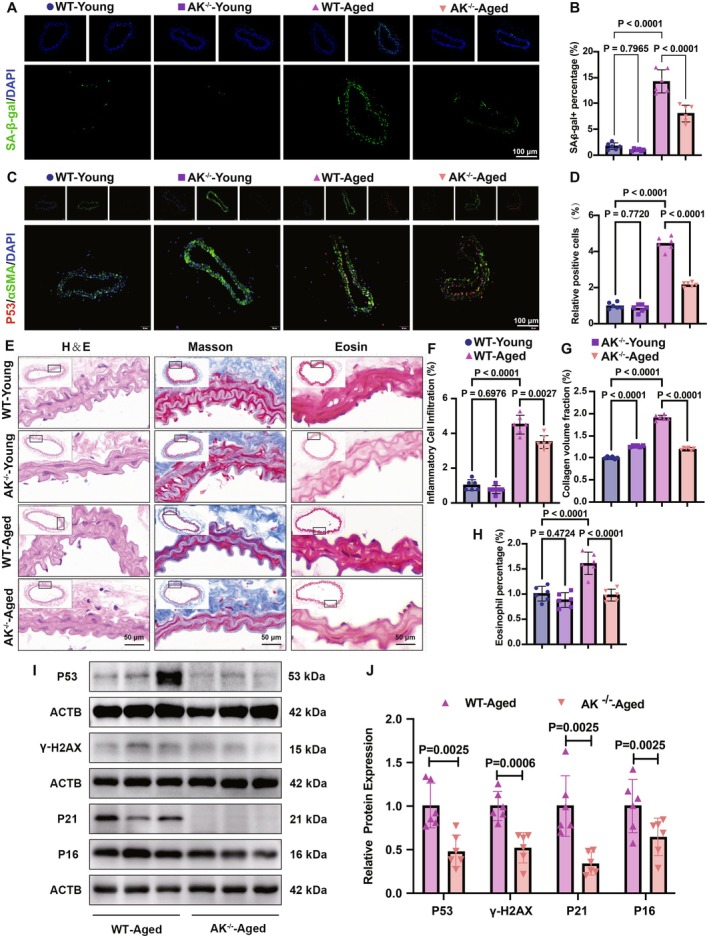
Knockout of AK050834 prevents vascular aging in mice in vivo. (A, B) Representative images (A) and quantitative analysis (B) of senescence‐associated β‐galactosidase (SA‐β‐gal) staining in aortic sections from WT and AK050834 knockout (AK−/−) mice in young (8‐week‐old) and aged groups (18‐month‐old). Scale bars, 100 μm. *n* = 6 per group. (C, D) Representative immunofluorescence images of p53 (red), α‐smooth muscle actin (α‐SMA, green), and DAPI (blue) staining (C), and quantification of p53‐positive cells (D) in aortic smooth muscle cells from the indicated groups. Scale bars, 100 μm. *n* = 6 per group. (E) Representative histopathological images of aortic sections stained with H&E, Masson's trichrome, and Eosin from the indicated groups. Scale bars, 50 μm. (F) Quantitative analysis of inflammatory cell infiltration based on H&E staining. *n* = 6 per group. (G) Quantitative analysis of collagen volume fraction based on Masson's trichrome staining. *n* = 6 per group. (H) Quantitative analysis of eosinophil percentage based on Eosin staining. *n* = 6 per group. (I) Western blot analysis of senescence‐associated proteins (P53, γ‐H2AX, P21, and P16) in aortic tissues from WT‐Aged (18‐month‐old) and AK−/− aged mice (18‐month‐old), with ACTB used as the internal control. Molecular weights are indicated. (J) Quantitative analysis of the relative protein expression levels of P53, γ‐H2AX, P21, and P16 based on the Western blot results from panel I. *n* = 6 per group. Data are presented as mean ± SEM. Comparisons between two groups (panel J) were analyzed by Student's *t*‐test. Comparisons among multiple groups (panels B, D, F, G, H) were analyzed by one‐way analysis of variance (ANOVA) followed by Tukey's post hoc test. Exact *p*‐values are indicated in the respective panels. Scale bars are indicated in the respective images.

**5. Knockout AK050834 improves age‐related cell dysfunction of proliferation and migration, cell cycle progression in VSMCs**.

Cells with senescence could result in dysfunction in proliferation, migration, and cell cycle progression (Thongon et al. 2021). To assess whether AK deficiency could alleviate D‐galactose (D‐gal)‐induced functional impairment, we isolated primary aortic smooth muscle cells (VSMCs) from WT and AK−/− mice and subjected them to D‐gal treatment. VSMCs were identified via immunofluorescence, with a purity exceeding 95% (Figure S5). Cell viability assays using the CCK‐8 method revealed that while D‐gal significantly reduced cell viability in WT cells, AK deficiency markedly restored the viability of VSMCs under D‐gal treatment (Figure 5A). Consistent with this, EdU staining demonstrated that the D‐gal‐induced reduction in cell proliferation was significantly reversed in AK−/− VSMCs (Figure 5B,C). Next, we evaluated cell migration capacity using Transwell chamber and scratch wound healing assays. The results showed that AK deficiency significantly rescued the D‐gal‐induced impairment of migratory capacity, as evidenced by increased numbers of migrated cells and enhanced wound healing rates in the AK−/− + D‐gal group compared to the WT + D‐gal group (Figure 5D–G). Furthermore, flow cytometry analysis of the cell cycle revealed that D‐gal treatment led to G0/G1 phase arrest in WT VSMCs, whereas AK deficiency effectively alleviated this arrest and promoted G1‐to‐S phase transition (Figure 5H). Mechanistically, Western blot analysis of key cell cycle regulators showed that the D‐gal‐induced downregulation of Cyclin D1 was significantly reversed by AK deficiency, while levels of Cyclin A2 and Cyclin B1 showed partial but less pronounced recovery (Figure 5I–L). Collectively, these findings indicate that AK deficiency alleviates D‐gal‐induced VSMC senescence‐associated dysfunction by restoring cell proliferation, promoting migration, and accelerating cell cycle progression.

**FIGURE 5:**
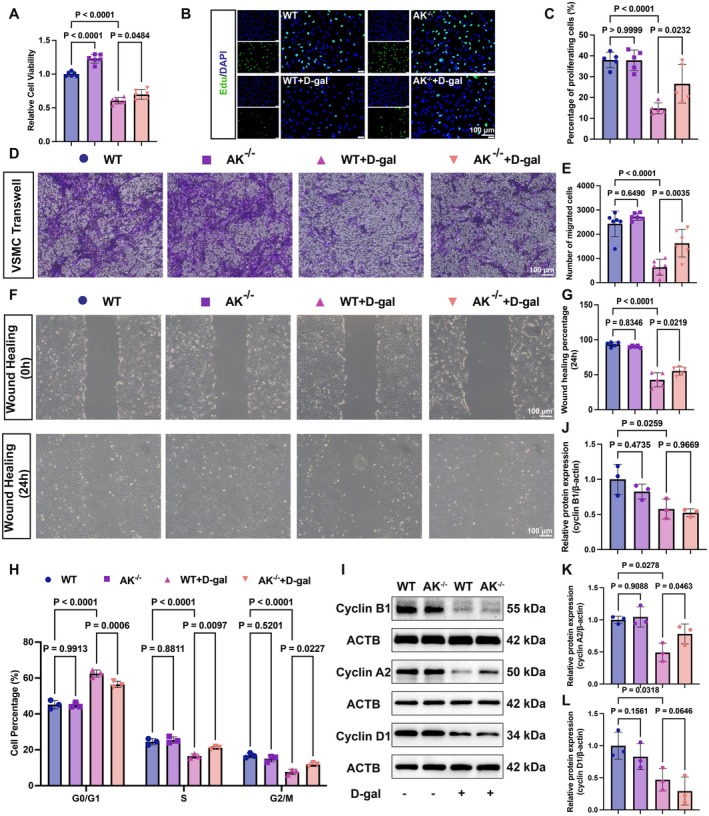
Knockout of AK050834 ameliorates D‐galactose‐induced dysfunction in cell proliferation, migration, and cell cycle progression in VSMCs. (A) Cell viability measured by CCK‐8 assay in VSMCs isolated from wild‐type (WT) and AK050834‐knockout (AK−/−) mice, with or without D‐galactose (D‐gal) treatment. *n* = 6 per group. (B, C) Representative EdU staining images (green) with DAPI (blue) counterstain (B) and quantitative analysis of the percentage of proliferating cells (C) in the indicated groups. Scale bar, 100 μm. *n* = 5 per group. (D, E) Representative images (D) and quantitative analysis of the number of migrated cells (E) evaluated by Transwell chamber assay in the indicated groups. Scale bar, 100 μm. *n* = 6 per group. (F, G) Representative images of wound healing assay at 0 h and 24 h (F), and quantitative analysis of the wound healing percentage at 24 h (G). Scale bar, 100 μm. *n* = 5 per group. (H) Quantitative analysis of cell cycle distribution (G0/G1, S, and G2/M phases) determined by flow cytometry with PI staining in the indicated groups. *n* = 3 per group. (I) Western blot analysis of key cell cycle regulators (Cyclin B1, Cyclin A2, and Cyclin D1) and ACTB (internal control) in VSMCs from the indicated groups, with molecular weights indicated. (J–L) Quantitative analysis of relative protein expression levels of Cyclin B1 (J), Cyclin A2 (K), and Cyclin D1 (L) based on the Western blot results from panel I. *n* = 3 per group. Data are presented as mean ± SEM. Comparisons among multiple groups were analyzed by one‐way analysis of variance (ANOVA) followed by Tukey's post hoc test. Exact *p*‐values are indicated in the respective panels. Scale bars are indicated in the respective images.

**6. RNA sequencing analysis indicates that ATF3 is a downstream target gene regulated by the Long non‐coding RNA AK050834 in controlling aortic smooth muscle function**.

To elucidate the mechanism by which lncRNA AK050834 regulates aortic vascular function, high‐throughput RNA sequencing analysis was performed on the aortas of WT and AK‐KO mice. A comparative analysis of the sequencing results revealed 490 differentially expressed genes (DEGs) following AK knockout, with 292 genes significantly upregulated and 198 genes downregulated in the experimental group (Figure 6A). Subsequent intersection of DEGs with those associated with vascular function facilitated GO, KEGG, and GSEA enrichment analyzes. GO results indicated significant enrichment of DEGs in a range of biological processes, including smooth muscle cell migration, cell senescence, apoptosis, proliferation, differentiation, and cell cycle regulation (Figure 6B). KEGG analysis revealed significant enrichment of DEGs in pathways including cellular senescence and the cell cycle (Figure 6C). GSEA results further demonstrated significant enrichment of DEGs in processes including cellular senescence, apoptosis, and the cell cycle (Figure 6D). In order to elucidate the target genes regulated by AK in the process of cellular senescence, the DEGs were intersected with a curated set of cellular senescence‐associated genes. This revealed that 17 of the 490 DEGs were highly correlated with cellular senescence (Figure 6E). The expression patterns of these 17 genes were visualized via a heatmap, which revealed that 5 target genes (including Cdkn1a) were downregulated and 12 target genes (including Cdk1, Atf3, and Serpine1) were upregulated in AK‐KO aortas (Figure 6F).

**FIGURE 6:**
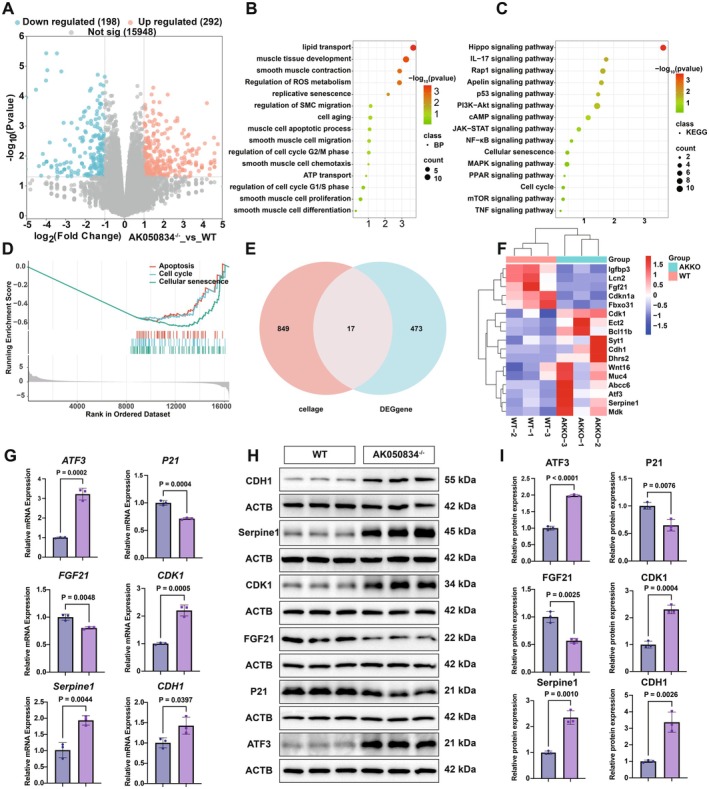
RNA‐seq analysis identifies ATF3 as a key downstream target gene of AK050834 in aortic smooth muscle cells. (A) Volcano plot illustrating the differentially expressed genes (DEGs) in aortic tissues of AK050834‐knockout (AK−/−) mice compared to wild‐type (WT) controls. Red dots indicate significantly upregulated genes (*n* = 292), blue dots indicate significantly downregulated genes (*n* = 198), and gray dots represent genes with no significant differences. *n* = 3 per group. (B, C) Functional enrichment analysis of DEGs. (B) Gene Ontology (GO) biological process enrichment analysis showing significant enrichment in terms associated with smooth muscle cell migration, proliferation, senescence, apoptosis, differentiation, and cell cycle regulation. (C) KEGG pathway enrichment analysis identifying significant enrichment in cellular senescence, cell cycle, and other signaling pathways. (D) Gene Set Enrichment Analysis (GSEA) confirming the significant enrichment of DEGs in cellular senescence, apoptosis, and cell cycle processes. (E) Venn diagram demonstrating the overlap between the 490 identified DEGs and a curated set of cellular senescence‐related genes, identifying 17 key senescence‐associated DEGs. (F) Heatmap displaying the expression patterns of the 17 senescence‐associated DEGs in AK−/− mice compared to WT controls. The color scale represents *Z*‐score normalized expression values. (G) Relative mRNA expression levels of ATF3, P21, FGF21, CDK1, Serpine1, and CDH1 validated by RT‐qPCR in aortic tissues from WT and AK−/− mice. *n* = 3 per group. (H) Western blot analysis of the indicated proteins (CDH1, Serpine1, CDK1, FGF21, P21, and ATF3) in aortic tissues from WT and AK−/− mice, with ACTB used as the internal control. Molecular weights are indicated. (I) Quantitative analysis of the relative protein expression levels of ATF3, P21, FGF21, CDK1, Serpine1, and CDH1 based on the Western blot results from panel H. *n* = 3 per group. DEGs were defined by |log_2_ (fold change)| > 1 and an adjusted *p*‐value < 0.05. Data are presented as mean ± SEM. Comparisons between two groups (panels G and I) were analyzed by Student's *t*‐test. Exact *p*‐values are indicated in the respective panels.

To validate the sequencing results at the molecular level, we examined the mRNA and protein expression levels of six representative senescence‐associated DEGs (ATF3, P21, FGF21, CDK1, Serpine1, and CDH1) in aortic tissues from WT and AK‐KO mice. RT‐qPCR analysis confirmed that AK knockout significantly upregulated the mRNA expression of ATF3, CDK1, Serpine1, and CDH1, while significantly downregulating the expression of P21 and FGF21 (Figure 6G). Consistent with the mRNA data, Western blot analysis further confirmed that AK knockout significantly increased the protein expression levels of ATF3, CDK1, Serpine1, and CDH1, while significantly reducing the levels of P21 and FGF21 in aortic tissues (Figure 6H), with quantitative analysis revealing robust statistical significance across all the tested proteins (Figure 6I). Collectively, these findings validate the sequencing data in vivo and confirm that AK deficiency bi‐directionally alters the expression profiles of multiple senescence‐associated genes, identifying ATF3, CDK1, Serpine1, CDH1, P21, and FGF21 as critical downstream effectors in the regulation of vascular aging.

**7. ATF3 knockdown exacerbates D‐galactose‐induced VSMC senescence, while ATF3 overexpression alleviates it**.

Given that RNA‐seq analysis identified ATF3 as one of the most significantly upregulated senescence‐associated genes in AK‐KO aortas (Figure 6), and given the established role of ATF3 as a pivotal transcription factor in cellular stress responses and senescence, we hypothesized that ATF3 serves as a critical downstream effector of AK in regulating VSMC senescence. To validate this, we conducted loss‐of‐function and gain‐of‐function assays in HA‐VSMCs. First, ATF3 was knocked down using specific siRNA (siATF3). SA‐β‐gal staining revealed that ATF3 knockdown significantly exacerbated D‐galactose (D‐gal)‐induced cellular senescence, as evidenced by a higher percentage of SA‐β‐gal‐positive cells in the siATF3 + D‐gal group compared with the siScramble+D‐gal group (Figure 7A,B). Consistent with this, Western blot analysis further demonstrated that ATF3 knockdown significantly upregulated the expression of P53, P21, P16, and γ‐H2AX under D‐gal treatment (Figure 7C,D).

**FIGURE 7:**
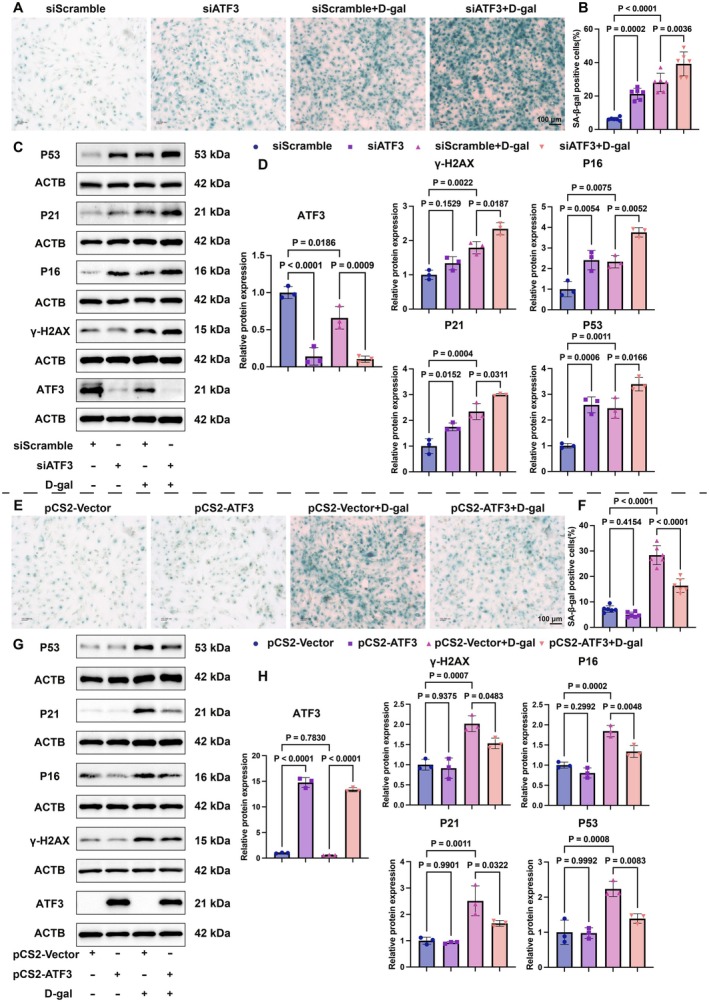
ATF3 knockdown exacerbates D‐galactose‐induced VSMC senescence, while ATF3 overexpression alleviates it. (A, B) Representative images (A) and quantitative analysis (B) of SA‐β‐gal staining in HA‐VSMCs transfected with scrambled siRNA (siScramble) or ATF3‐specific siRNA (siATF3), followed by treatment with or without D‐galactose (D‐gal). Scale bar, 100 μm. *n* = 6 per group. (C, D) Western blot analysis of senescence‐associated proteins (P53, P21, P16, γ‐H2AX) and ATF3 (C), and quantification of relative protein expression levels (D) in HA‐VSMCs from the indicated groups. ACTB was used as the internal control. Molecular weights are indicated. *n* = 3 per group. (E, F) Representative images (E) and quantitative analysis (F) of SA‐β‐gal staining in HA‐VSMCs transfected with empty vector (pCS2‐Vector) or ATF3 overexpression plasmid (pCS2‐ATF3), followed by treatment with or without D‐galactose (D‐gal). Scale bar, 100 μm. *n* = 6 per group. (G, H) Western blot analysis of senescence‐associated proteins (P53, P21, P16, γ‐H2AX) and ATF3 (G), and quantification of relative protein expression levels (H) in HA‐VSMCs from the indicated groups. ACTB was used as the internal control. Molecular weights are indicated. *n* = 3 per group. Data are presented as mean ± SEM. Comparisons among multiple groups were analyzed by one‐way analysis of variance (ANOVA) followed by Tukey's post hoc test. Exact *p*‐values are indicated in the respective panels. Scale bars are indicated in the respective images.

Conversely, overexpression of ATF3 (pCS2‐ATF3) significantly reversed the D‐gal‐induced senescent phenotype. The percentage of SA‐β‐gal‐positive cells in the pCS2‐ATF3 + D‐gal group was markedly lower than that in the pCS2‐Vector+D‐gal group (Figure 7E,F). Furthermore, Western blot analysis confirmed that ATF3 overexpression significantly suppressed the D‐gal‐induced upregulation of P53, P21, P16, and γ‐H2AX proteins (Figure 7G,H). Collectively, these results indicate that ATF3 functions as a critical negative regulator of D‐gal‐induced VSMC senescence, as evidenced by the fact that ATF3 deficiency accelerates the senescent phenotype, whereas its overexpression effectively alleviates it.

**8. The Long non‐coding RNA AK050834 suppresses ATF3 activity by binding to its promoter region and stabilizes p21 mRNA**.

Having identified ATF3 as a significantly upregulated senescence‐associated gene upon AK deletion in our RNA‐seq data, and given its established role as a pivotal transcription factor in cellular stress and senescence, we hypothesized that AK drives VSMC senescence by directly inhibiting ATF3 expression. Furthermore, considering the nuclear localization of AK and the observed concomitant downregulation of P21 in the sequencing results, we speculated that AK may exert dual regulatory functions: not only repressing ATF3 transcription but also modulating P21 expression at the post‐transcriptional level via mRNA stabilization.

To validate the former mechanism, we first confirmed that AK knockout significantly upregulated Atf3 mRNA and protein expression in primary mouse aortic smooth muscle cells (VSMCs) by RT‐qPCR and Western blotting, respectively (Figure 8A–C). Given the nuclear localization of AK, we next investigated whether AK directly represses ATF3 transcriptionally. Prediction analysis identified two potential binding sites (Site 1 and Site 2) within the ATF3 promoter region (from −2000 bp to 0 bp) (Figure 8D). Luciferase reporter assays revealed that AK overexpression significantly inhibited the activity of the full‐length ATF3 promoter and the Site 1 construct, but did not affect Site 2 (Figure 8E). To validate the direct physical interaction, we performed chromatin isolation by RNA purification (ChIRP) assays, which confirmed that AK was significantly enriched at Site 1, but not at Site 2 (Figure 8F). Furthermore, mutating the Site 1 sequence completely abolished the inhibitory effect of AK on promoter activity (Figure 8G), collectively demonstrating that AK suppresses ATF3 transcription by directly binding to the Site 1 region of its promoter.

**FIGURE 8:**
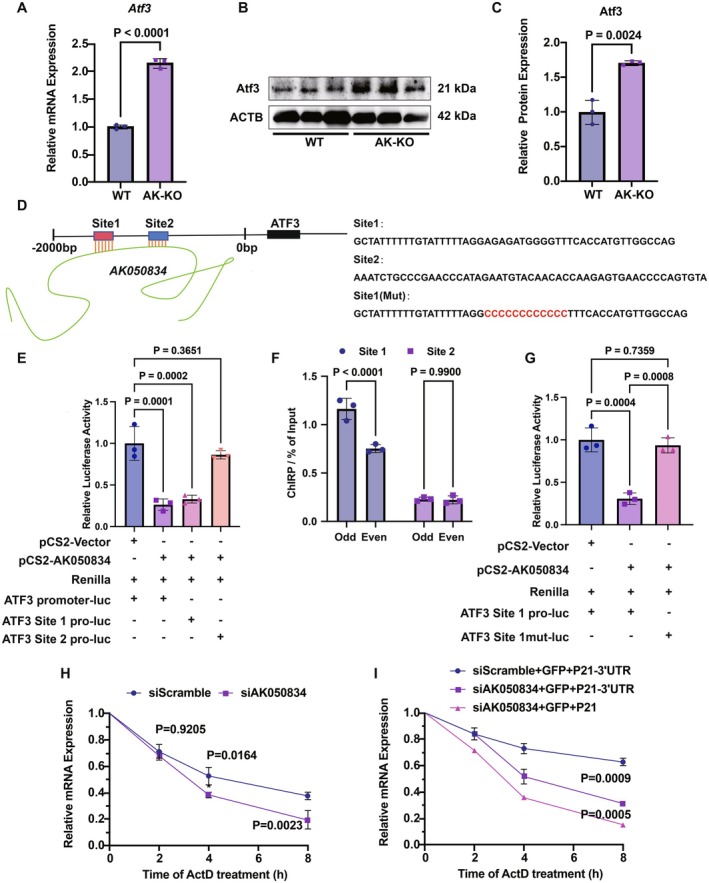
LncRNA AK050834 suppresses ATF3 promoter activity and stabilizes p21 mRNA to regulate vascular smooth muscle cell senescence. (A) Relative mRNA expression of Atf3 in primary aortic smooth muscle cells (VSMCs) isolated from wild‐type (WT) and AK050834‐knockout (AK‐KO) mice, measured by RT‐qPCR. *n* = 3 per group. (B, C) Representative Western blot images (B) and quantitative analysis of relative ATF3 protein expression (C) in primary VSMCs from WT and AK‐KO mice, with ACTB used as the internal control. *n* = 3 per group. (D) Schematic diagram of the ATF3 promoter region (−2000 bp to 0 bp), illustrating two predicted AK050834 binding sites (Site 1 and Site 2) and the specific mutant sequence for Site 1 (Site1 Mut) used in subsequent experiments. (E) Luciferase reporter assays evaluating the activity of the full‐length ATF3 promoter (ATF3 promoter‐luc), Site 1 (ATF3 Site 1 pro‐luc), and Site 2 (ATF3 Site 2 pro‐luc) constructs in HEK‐293 T cells co‐transfected with pCS2‐Vector or pCS2‐AK050834. *n* = 3 per group. (F) Chromatin isolation by RNA purification (ChIRP) followed by qPCR assessing the binding enrichment of AK050834 to Site 1 and Site 2 within the ATF3 promoter. *n* = 3 per group. (G) Luciferase reporter assay using the wild‐type Site 1 construct and the mutant Site 1 construct (ATF3 Site 1 mut‐luc) in HEK‐293 T cells co‐transfected with pCS2‐Vector or pCS2‐AK050834. *n* = 3 per group. (H) Actinomycin D (ActD) chase assay evaluating the mRNA stability of p21 in HA‐VSMCs transfected with control siRNA (siScramble) or AK050834‐specific siRNA (siAK050834). Relative mRNA expression was measured at the indicated time points after ActD treatment. *n* = 3 per group per time point. (I) Actinomycin D (ActD) chase assay assessing the stabilization of exogenous p21 mRNA in HEK‐293 T cells. Cells were co‐transfected with siScramble or siAK050834, together with a GFP + P21 construct with or without the 3′UTR region. Relative mRNA levels were measured at the indicated times. *n* = 3 per group per time point. Data are presented as mean ± SEM. Comparisons between two groups were analyzed by Student's *t*‐test. Comparisons among multiple groups were analyzed by one‐way analysis of variance (ANOVA) followed by Tukey's post hoc test. Exact *p*‐values are indicated in the respective panels.

To address the second mechanism, we explored whether AK could directly influence P21 mRNA stability. Actinomycin D chase assays revealed that knocking down AK significantly accelerated the degradation rate of endogenous P21 mRNA in HA‐VSMCs (Figure 8H), indicating that AK acts to stabilize P21 mRNA. To investigate the structural basis of this stabilization, we expressed exogenous P21 mRNA with or without the 3′ untranslated region (3′UTR) under a CMV promoter. Notably, while the presence of the 3′UTR conferred AK‐dependent stability, its removal led to a further significant reduction in P21 mRNA stability upon AK knockdown (Figure 8I). Given that the 3′UTR serves as the primary binding platform for RNA‐binding proteins (RBPs) and regulatory non‐coding RNAs that dictate mRNA turnover, these observations strongly indicate that AK stabilizes P21 mRNA by interacting with its 3′UTR. The enhanced degradation observed in the 3′UTR‐deficient constructs is likely due to the loss of this critical cis‐regulatory platform, rendering the transcript intrinsically vulnerable to exonucleolytic decay and simultaneously removing the protective effect conferred by AK. While the precise RBPs responsible for mediating AK‐induced P21 mRNA stabilization require further identification, our data suggest that AK likely functions as a scaffold to recruit distinct RBPs to the 3′UTR of P21, thereby preventing its degradation and contributing to the senescent phenotype.

Collectively, these data reveal a dual regulatory model in which AK drives VSMC senescence by not only inhibiting ATF3 promoter activity through direct binding to Site 1 but also independently enhancing P21 expression through 3′UTR‐dependent mRNA stabilization, likely via an RBP‐mediated mechanism. Exact *p*‐values are indicated in the respective panels.

**9. Overexpression of ATF3 rescues AK050834‐induced VSMC senescence**.

To definitively validate the hypothesis that AK drives VSMC senescence by suppressing ATF3, rescue experiments were conducted by co‐transfecting HA‐VSMCs with AK‐overexpressing (pCS2‐AK) and ATF3‐overexpressing (pCMV‐ATF3) plasmids. SA‐β‐gal staining revealed that overexpression of AK led to a significant increase in the proportion of SA‐β‐gal‐positive cells compared with the control group. However, co‐transfection with ATF3 markedly reduced this proportion, restoring it to near‐normal levels (Figure 9A,B), indicating that ATF3 effectively counteracts the cellular senescence induced by AK.

**FIGURE 9:**
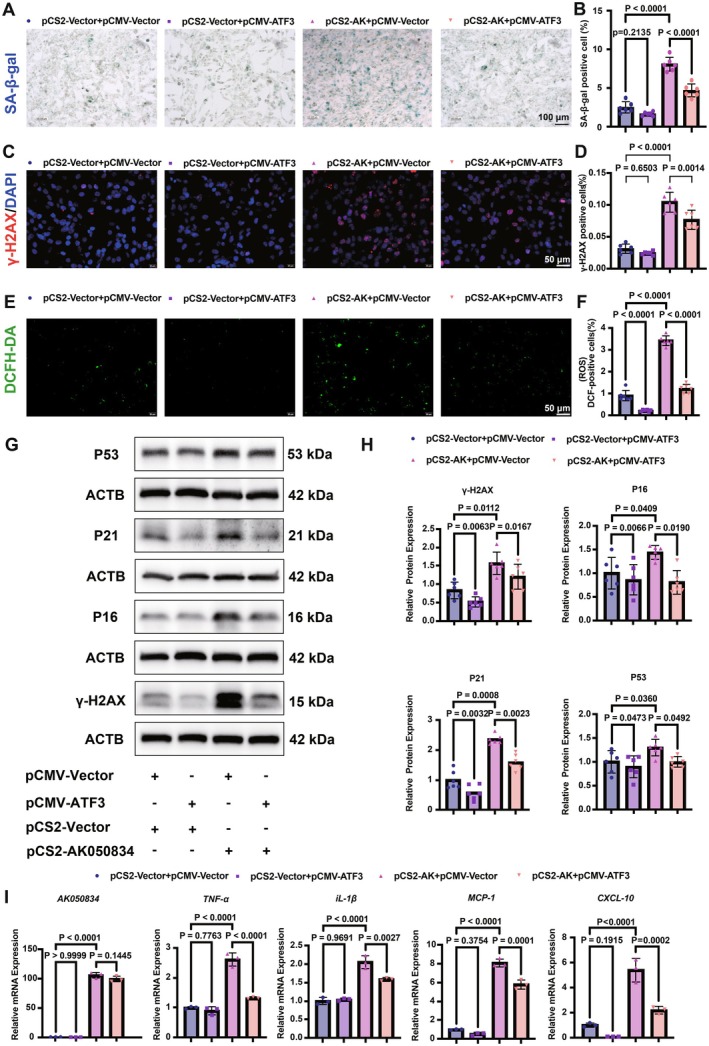
Overexpression of ATF3 rescues AK050834‐induced senescence in human aortic VSMCs. (A, B) Representative images (A) and quantitative analysis (B) of SA‐β‐gal staining in HA‐VSMCs co‐transfected with empty vectors (pCS2‐Vector+pCMV‐Vector), pCMV‐ATF3 alone (pCS2‐Vector+pCMV‐ATF3), AK050834 overexpression plasmid alone (pCS2‐AK + pCMV‐Vector), or co‐transfected with both plasmids (pCS2‐AK + pCMV‐ATF3). Scale bar, 100 μm. *n* = 6 per group. (C, D) Representative immunofluorescence images of γ‐H2AX (red) with DAPI (blue) staining (C) and quantification of γ‐H2AX‐positive nuclei (D) in the indicated groups. Scale bar, 50 μm. *n* = 6 per group. (E, F) Representative images of DCFH‐DA fluorescence (green) for detecting ROS levels (E) and the percentage of ROS‐positive cells (F) in the indicated groups. Scale bar, 50 μm. *n* = 6 per group. (G) Western blot analysis of senescence‐associated proteins (P53, P21, P16, and γ‐H2AX) in HA‐VSMCs from the indicated groups. ACTB was used as the internal control. (H) Quantitative analysis of the relative protein expression levels of γ‐H2AX, P16, P21, and P53 based on the Western blot results from panel G. *n* = 6 per group. (I) Relative mRNA expression levels of AK050834 and senescence‐associated secretory phenotype (SASP) factors (TNF‐α, IL‐1β, MCP‐1, and CXCL‐10) determined by RT‐qPCR in the indicated groups. *n* = 3 per group. Data are presented as mean ± SEM. Comparisons among multiple groups were analyzed by one‐way analysis of variance (ANOVA) followed by Tukey's post hoc test. Exact *p*‐values are indicated in the respective panels. Scale bars are indicated in the respective images.

Next, we assessed DNA damage and oxidative stress levels. Immunofluorescence staining revealed that VSMCs overexpressing AK exhibited a significant increase in γ‐H2AX foci and ROS fluorescence signals. Conversely, the overexpression of ATF3 led to a substantial decrease in both γ‐H2AX foci and ROS levels (Figure 9C–F). Furthermore, Western blot analysis demonstrated that AK overexpression significantly upregulated the expression of senescence‐associated proteins P53, P21, P16, and γ‐H2AX. Importantly, ATF3 co‐expression effectively reversed these changes, leading to a marked reduction in the expression levels of these proteins (Figure 9G,H). RT‐qPCR results further validated this trend at the mRNA level (Figure S6). Additionally, assessment of senescence‐associated secretory phenotype (SASP) factors revealed that AK overexpression significantly promoted the mRNA expression of TNF‐α, IL‐1β, MCP‐1, and CXCL‐10. Notably, this upregulation of pro‐inflammatory factors was largely suppressed by ATF3 overexpression (Figure 9I).

Collectively, these rescue experiments provide definitive cellular evidence demonstrating that AK050834 drives VSMC senescence via the suppression of ATF3. Our findings establish that while AK overexpression triggers DNA damage, oxidative stress, and an exacerbated SASP response to promote a comprehensive senescent phenotype, exogenous ATF3 supplementation effectively halts this pathological cascade, significantly reversing the induction of classic senescence markers. These results highlight the functional centrality of the AK‐ATF3 regulatory axis in vascular aging and suggest that targeting ATF3 activation may represent a potential therapeutic strategy against AK‐mediated vascular senescence and associated cardiovascular disorders.

## 4 Discussion

This study systematically elucidates, for the first time, the pivotal role of the AK in vascular aging and its underlying molecular mechanisms. Our research reveals that AK exhibits high expression in vascular tissues and is primarily localized within the cell nucleus, providing crucial evidence for its potential role in epigenetic regulation in vessels. Recent studies have indicated that nuclear‐localized lncRNAs typically influence gene expression through mechanisms such as chromatin remodeling, transcriptional regulation, or promoter interactions (Yi et al. 2014). In accordance with the findings of this study, many investigations have demonstrated that nuclear lncRNAs can function as molecular platforms, thereby recruiting chromatin‐modifying complexes to specific genomic regions and thus regulating the transcriptional activity of downstream genes (Ma et al. 2018). The nuclear localization of AK suggests the possibility of its direct participation in the regulation of the transcription of aging‐related genes in aortic VSMCs.

It is noteworthy that the subcellular localization of lncRNAs is closely linked to their functional roles (Li et al. 2021). It has been demonstrated that nuclear lncRNAs, including XIST and HOTAIR, have the capacity to influence cell fate decisions by regulating histone modifications (Chu et al. 2015; Tsai et al. 2010). The current study provides a novel classification of AK as a nuclear regulatory ncRNA, offering a new perspective on the understanding of the spatially specific functions of such non‐coding RNAs in vascular aging.

A salient finding of this study is that AK deficiency enhances the proliferation and migration of VSMCs, while concomitantly accelerating cell cycle progression. This effect provides compelling evidence for the involvement of AK in vascular senescence phenotypes. In aged VSMCs, cells typically exhibit proliferative arrest, diminished migratory capacity, and cell cycle blockage (Gnani et al. 2019); AK knockout effectively reversed these aging‐associated phenotypes. It is particularly noteworthy that both in vivo and in vitro experiments demonstrated that AK knockout or knockdown effectively mitigated vascular senescence, whereas overexpression of AK in vitro exacerbated senescent phenotypes. This dual validation provides compelling evidence for the causal role of AK in vascular aging.

Phenotypic switching in aortic smooth muscle cells constitutes a pivotal step in vascular aging (Yoshida et al. 2008; Clément et al. 2019). Under physiological conditions, VSMCs maintain a contractile phenotype, whereas pathological stimuli induce a shift toward a synthetic phenotype characterized by increased proliferation, migration, and inflammatory cytokine secretion. The present findings suggest that AK may function as a pivotal regulator of this phenotypic switch. Mechanical stress, a significant contributor to vascular aging, has been shown to impact VSMC function by regulating the expression of lncRNAs (Lacolley et al. 2017). The present study may establish a novel molecular link between mechanical stress and epigenetic regulation in vascular aging.

Through the utilization of RNA sequencing analysis, we were able to identify ATF3 as a pivotal downstream target gene that is regulated by AK in the context of VSMC function. As a stress response transcription factor, ATF3 plays a crucial role in cell cycle regulation and the aging process (Liu et al. 2023; Wen et al. 2024; Fu et al. 2021). It is important to note that the functional role of ATF3 in cellular senescence and tissue aging is highly context‐dependent. While ATF3 has been reported to promote senescence in non‐vascular cell types such as macrophages (Wang et al. 2021; Deng et al. 2026), osteocytes (Han et al. 2026; Yin et al. 2024), and periodontal ligament cells (Bae and Lee 2025) under specific stress conditions, recent studies have uncovered a distinct and protective role for ATF3 within the vascular bed. Specifically, previous evidence has demonstrated that ATF3 deficiency exacerbates age‐related atherosclerosis, a process intimately linked to the acceleration of VSMC senescence (Nie et al. 2025). This pro‐arteriosclerotic effect driven by ATF3 deficiency aligns well with our own findings in the D‐gal‐induced VSMC senescence model. Our complementary loss‐of‐function and gain‐of‐function experiments clearly demonstrated that ATF3 overexpression effectively rescued D‐gal‐induced VSMC senescence, whereas ATF3 knockdown exerted the opposite effect. Collectively, integrating our results with the aforementioned atherosclerosis evidence strongly corroborates that, within the specific context of VSMCs and vascular aging, ATF3 functions as a critical negative regulator of senescence. Therefore, ATF3 acts as an intrinsic “brake” against VSMC senescence, and its deficiency removes this protective barrier, thereby rendering the vascular wall more susceptible to aging‐driven pathological remodeling.

Our mechanistic investigations further revealed that AK directly binds to the ATF3 promoter region to inhibit its activity while simultaneously stabilizing p21 mRNA. This dual role as both a pro‐aging and an anti‐aging gene is a novel discovery in aging research and non‐coding RNA studies. The results demonstrate that AK suppresses ATF3 transcriptional activity by directly binding to its promoter region. This finding is highly consistent with contemporary research focusing on the mechanisms through which lncRNAs modulate gene expression. A plethora of evidence has demonstrated that lncRNAs regulate gene transcription by forming RNA–DNA triplexes with promoter regions or by recruiting inhibitory complexes (Leisegang et al. 2024). As a pivotal mediator of cellular stress responses, reduced ATF3 expression has been shown to be closely associated with various aging‐related pathological processes. The present findings establish the first link between the regulation of a lncRNA and the functional role of ATF3 in vascular aging, thereby enriching our understanding of the transcriptional regulatory network that governs vascular aging.

Our study indicates that AK enhances the stability of p21 mRNA. As a classic cyclin‐dependent kinase inhibitor, p21 accumulation constitutes a hallmark event of cellular senescence (Nie et al. 2024). Recent studies have highlighted that the regulation of mRNA stability is a critical component of gene expression control, with lncRNAs modulating mRNA half‐lives either through direct binding or by recruiting RNA‐binding proteins (RBPs) (Zhao et al. 2018; Yu et al. 2018). Our actinomycin D chase assays demonstrated that AK knockdown significantly accelerated the degradation of p21 mRNA, and the removal of the 3′UTR region further compromised its stability. Given that the 3′UTR serves as the primary binding platform for RBPs dictating mRNA turnover, we propose that AK may act as a molecular scaffold to recruit specific RBPs to the 3′UTR of p21, thereby protecting it from exonucleolytic degradation. Although the precise RBPs involved in this process warrant further identification through future approaches such as RNA pull‐down coupled with mass spectrometry (MS), our findings offer novel insights into the lncRNA‐mediated post‐transcriptional regulation of senescence‐associated genes through RBP‐dependent mechanisms.

Notably, ATF3 and p21 exhibit close interactions in cell cycle regulation. Previous studies have demonstrated that ATF3 can transcriptionally suppress p21 expression (Nie et al. 2025). In light of our findings, we propose that AK orchestrates a dual synergistic regulatory mechanism in driving VSMC senescence: at the transcriptional level, AK directly binds to the ATF3 promoter to inhibit its activity, thereby relieving the ATF3‐mediated transcriptional repression of p21; meanwhile, at the post‐transcriptional level, AK stabilizes p21 mRNA via its 3′UTR. This dual, synergistic action—combining the relief of transcriptional repression with the enhancement of mRNA stability—potently drives the accumulation of p21 protein, thereby facilitating the precise and robust progression of VSMC senescence.

The regulatory network identified in this study, AK‐ATF3‐p21, provides a novel theoretical framework for the epigenetic mechanisms of vascular aging. From a theoretical perspective, the present study contributes to the expansion of the current understanding of the functions of lncRNAs in vascular biology. The study proposes a novel paradigm whereby these non‐coding RNAs influence vascular aging through the simultaneous regulation of transcriptional and post‐transcriptional processes. This finding is consistent with current research trends that emphasize the significance of epigenetic regulation in the process of vascular aging.

From a translational medicine perspective, the potential practical applications of our research are significant. Firstly, it is proposed that AK may serve as a novel biomarker for vascular aging, thereby enabling the early identification of individuals at high risk of vascular senescence. Secondly, targeting AK has the potential to yield novel therapeutic strategies for the retardation of vascular aging.

It is noteworthy that the present findings demonstrate that the upregulation of ATF3 results in resistance to the deleterious effects of AK‐induced vascular aging. This finding not only validates the pivotal role of ATF3 downstream of AK, but also suggests that ATF3 agonists or activation strategies may represent potential therapeutic approaches against vascular aging.

In summary, this study systematically unveils a novel pathway through which lncRNA AK regulates vascular aging via dual opposite regulation of two senescence‐sensitive genes, ATF3 and p21. These findings not only deepen our understanding of the epigenetic mechanisms underlying vascular aging, but also provide theoretical foundations and potential targets for developing innovative strategies to delay vascular aging.

## 5 Limitations of the Study

Despite the fact that this study systematically elucidates the pivotal role of AK in vascular senescence, certain limitations remain. Firstly, further investigation is required into the precise molecular mechanism by which AK recognizes the ATF3 promoter region. It is imperative that further exploration be undertaken in order to ascertain whether this pertains to specific sequence recognition or protein‐mediated indirect effects. Secondly, the precise mechanism by which AK stabilizes p21 mRNA remains to be elucidated. The question of whether this phenomenon occurs through direct binding or by regulating RNA‐binding protein activity also remains to be elucidated and requires further investigation. Future research may focus on the following directions: firstly, the utilization of CHIRP‐MS or RNA pull‐down combined with mass spectrometry to identify the interaction protein network of AK; secondly, the exploration of the function of AK in other vascular cell types, such as endothelial cells; thirdly, the investigation of the correlation between AK expression and age in human vascular tissues; and fourthly, the development of intervention strategies targeting AK and the evaluation of their therapeutic potential.

## Author Contributions

Chunxiang Zhang and Haoran Wu designed and led this research project. Haoran Wu, Ping Yang, Yang Liu, and Qiuting Li conducted experiments and analyzed data. Xinglin Wang, Linyi Tang, Liyun Li, Ting Huang, and Xin Zhang conducted experiments. Qiuting Li contributed reagents or other essential materials. Chunxiang Zhang and Haoran Wu wrote the paper and provided research funding.

## Funding

This work was supported by National Natural Science Foundation of China (Grant U23A20398), Noncommunicable Chronic Diseases‐National Science and Technology Major Project (Grant 2024ZD0537707), Sichuan Science and Technology Program (Grants 2025YFRG0005 and 2025ZNSFSC0662), Research Start‐up Foundation of Southwest Medical University (Grant 00040155), Institutional Scientific Research Project of Southwest Medical University (Grant 40/00160741), Luzhou Science and Technology Program (Grant 2023JYJ003), Health Commission of Sichuan Province Medical Science and Technology Program (Grant 25QNMP060), Chengdu University of Traditional Chinese Medicine 2025 Joint University‐Faculty Innovation Fund (Grant XYLH202501024), and Research Special Project of Sichuan Administration of Traditional Chinese Medicine (Grant 26MSZX375).

## Conflicts of Interest

The authors declare no conflicts of interest.

## Supporting information


**Figure S1:** Sequence alignment showing high homology (91.4%) of lncRNA AK050834 between human and mouse genomes, indicating strong evolutionary conservation.
**Figure S2:** Overexpression of lncRNA AK050834 exacerbates D‐galactose‐induced senescence in human aortic vascular smooth muscle cells. (A) RT‐qPCR detection of P53 expression (*n* = 12); (B) RT‐qPCR detection of P21 expression (*n* = 12); (C) RT‐qPCR detection of P16 expression (*n* = 12); Comparisons among multiple groups were analyzed by one‐way analysis of variance (ANOVA) followed by Tukey's post hoc test. Data represent mean ± SEM, *p < 0.05, **p < 0.01, ***p < 0.001. ns, no significant.
**Figure S3:** Knockdown of lncRNA AK050834 alleviates D‐galactose‐induced senescence in human aortic vascular smooth muscle cells. (A) RT‐qPCR detection of P53 expression (*n* = 12); (B) RT‐qPCR detection of P21 expression (*n* = 12); (C) RT‐qPCR detection of P16 expression (*n* = 12); Comparisons among multiple groups were analyzed by one‐way analysis of variance (ANOVA) followed by Tukey's post hoc test. Data represent mean ± SEM, *p < 0.05, **p < 0.01, ***p < 0.001. ns, no significant.
**Figure S4:** Systemic knockout of lncRNA‐AK050834 using CRISPR‐Cas9 technology. (A) Schematic diagram of sgRNA target sequences; (B) Identification of mouse tail genotypes using agarose gel electrophoresis.
**Figure S5:** Isolation of primary mouse aortic smooth muscle cells. (A) In vitro primary aortic smooth muscle cell migration under light microscopy; (B) Identification of primary aortic smooth muscle cells using α‐SMA immunofluorescence, with cell nuclei stained using DAPI.
**Figure S6:** Overexpression of ATF3 rescues lncRNA AK050834‐induced senescence in human aortic vascular smooth muscle cells. (A) RT‐qPCR detection of P53 expression (*n* = 3); (B) RT‐qPCR detection of P21 expression (*n* = 3); (C) RT‐qPCR detection of P16 expression (*n* = 3); Comparisons among multiple groups were analyzed by one‐way analysis of variance (ANOVA) followed by Tukey's post hoc test. Data represent mean ± SEM, *p < 0.05, **p < 0.01, ***p < 0.001. ns, no significant.
**Figure S7:** D‐galactose does not induce acute cytotoxicity in HA‐VSMCs at the concentration used for senescence induction. Cell viability was assessed by CCK‐8 assay in HA‐VSMCs treated with increasing concentrations of D‐galactose (0–40 g/L) for 24 h. The results revealed that D‐galactose treatment up to 40 g/L did not significantly affect cell viability compared with the control group (0 g/L), indicating that the 40 g/L concentration used in the senescence induction experiments does not cause acute cytotoxicity. Data represent mean ± SEM. *n* = 3 per group.

## Data Availability

The data that support the findings of this study are available on request from the corresponding author. The data are not publicly available due to privacy or ethical restrictions.
