# Supplementary material for: AK050834 Deficiency Prevents Vascular Aging via Opposite Regulation of Two Senescence‐Sensitive Genes ATF3 and p21
Source: Aging Cell. 2026 Aug 18;25(8):e70670. doi: 10.1111/acel.70670 (PMC13483296; doi:10.1111/acel.70670)
Supplement: Supplementary file 1 [file ACEL-25-e70670-s001.docx]

**Supplementary** **Figure S1.** Sequence alignment showing high homology (91.4%) of lncRNA AK050834 between human and mouse genomes, indicating strong evolutionary conservation.

**Supplementary Figure S2. Overexpression of lncRNA AK050834 exacerbates D-galactose-induced senescence in human aortic vascular smooth muscle cells**. (A) RT-qPCR detection of P53 expression(n=12); (B) RT-qPCR detection of P21 expression(n=12); (C) RT-qPCR detection of P16 expression(n=12); Comparisons among multiple groups were analyzed by one-way analysis of variance (ANOVA) followed by Tukey’s post-hoc test. Data represent mean±SEM, * *P* <0.05, ** *P* <0.01, *** *P* <0.001. ns: no significant.

**Supplementary Figure S3. Knockdown of lncRNA AK050834 alleviates D-galactose-induced senescence in human aortic vascular smooth muscle cells.** (A) RT-qPCR detection of P53 expression(n=12); (B) RT-qPCR detection of P21 expression(n=12); (C) RT-qPCR detection of P16 expression(n=12); Comparisons among multiple groups were analyzed by one-way analysis of variance (ANOVA) followed by Tukey’s post-hoc test. Data represent mean±SEM, * *P* <0.05, ** *P* <0.01, *** *P* <0.001. ns: no significant.

**Supplementary Figure S4. Systemic knockout of lncRNA-AK050834 using CRISPR-Cas9 technology.** (A) Schematic diagram of sgRNA target sequences;(B) Identification of mouse tail genotypes using agarose gel electrophoresis.

**Supplementary Figure S5. Isolation of primary mouse aortic smooth muscle cells**. (A) In vitro primary aortic smooth muscle cell migration under light microscopy;(B) Identification of primary aortic smooth muscle cells using α-SMA immunofluorescence, with cell nuclei stained using DAPI.

**Supplementary Figure S6. Overexpression of ATF3 rescues lncRNA AK050834-induced senescence in human aortic vascular smooth muscle cells****.** (A) RT-qPCR detection of P53 expression(n=3); (B) RT-qPCR detection of P21 expression(n=3); (C) RT-qPCR detection of P16 expression(n=3); Comparisons among multiple groups were analyzed by one-way analysis of variance (ANOVA) followed by Tukey’s post-hoc test. Data represent mean±SEM, * *P* <0.05, ** *P* <0.01, *** *P* <0.001. ns: no significant.

**Supplementary Figure S7. D-galactose does not induce acute cytotoxicity in HA-VSMCs at the concentration used for senescence induction.**

Cell viability was assessed by CCK-8 assay in HA-VSMCs treated with increasing concentrations of D-galactose (0–40 g/L) for 24 h. The results revealed that D-galactose treatment up to 40 g/L did not significantly affect cell viability compared with the control group (0 g/L), indicating that the 40 g/L concentration used in the senescence induction experiments does not cause acute cytotoxicity. Data represent mean ± SEM. n = 3 per group.
